# The Genistein Supply and Elemental Composition of Rat Kidneys in an Induced Breast Cancer Model

**DOI:** 10.3390/nu17071184

**Published:** 2025-03-28

**Authors:** Dorota Skrajnowska, Arkadiusz Szterk, Karol Ofiara, Paweł Kowalczyk, Barbara Bobrowska-Korczak

**Affiliations:** 1 Department of Toxicology and Food Science, Faculty of Pharmacy, Medical University of Warsaw, Banacha 1, 02-097 Warsaw, Poland; dorota.skrajnowska@wum.edu.pl (D.S.);; 2 ASLAB Science, Fort Służew 1/9, 02-787 Warsaw, Polandk.ofiara@aslabsci.com (K.O.); 3 Chair of Preclinical Sciences, Department of Pharmacology and Toxicology, Warsaw University of Life Sciences, Nowoursynowska 166, 02-787 Warsaw, Poland

**Keywords:** kidneys, genistein, trace elements, homeostasis

## Abstract

**Background:** Many natural phytochemicals support the work of the kidneys. The health effects of genistein have been confirmed in many kidney diseases (inflammation and acute kidney injury, cancer or menopausal or senile changes). Genistein through various mechanisms can affect kidney conditions. **Objectives:** The purpose of this work was to analyze the supply of various forms of genistein at a low dose (0.2 mg/kg b.w.) on the renal mineral composition of rats under conditions of mammary gland tumorigenesis (induced with DMBA). **Methods**: Sprague rats at the age of 40 days were divided into four research groups, i.e., a control group receiving only standard feed and four groups receiving feed supplemented with genistein in the form of nanoparticles (0.1 mg/mL, i.e., 0.2 mg/kg.i.d.) (size: 92 ± 41 nm), genistein in microparticle form (0.1 mg/mL, i.e., 0.2 mg/kg.i.d.) (size: 587 ± 83 nm) and genistein in macroparticle form (normal, classical) (0.1 mg/mL, i.e., 0.2 mg/kg.i.d.). Mammary gland cancer was induced using DMBA (7,12-dimethyl-1,2-benz(a)anthracene). The experiment lasted 100 days. The concentrations of Ca, Zn, Fe, Cu, As, Se, Rb, Sr, Mo, B, and Mn were measured using the ICP-MS method, while the levels of K, Mg, and Na were measured using the FAAS method. **Results**: It was shown that, depending on the degree of miniaturization of genistein, its administration affected changes in kidney mineral composition, primarily resulting in a strongly reduced calcium content in the group of rats receiving nanogenistein. We found a negative impact of nanogenistein administration on the amount of calcium and iron, indicating an increased distribution or excretion of these elements from the body, as well as an increase in the number of elements, especially magnesium, sodium, zinc, boron, and copper concentrations, compared to the non-supplemented group. **Conclusions**: This study confirms the need for thorough clinical analyses in the future, with regard to the effects of genistein, especially its nanoforms on the body.

## 1. Introduction

The literature is replete with data on attempts to use genistein in the treatment of osteoporosis, cancer, obesity, osteoarthritis, encephalopathy and retinopathy, type 2 diabetes and other diseases [1,2,3,4,5,6]. However, its low bioavailability associated with poor water solubility, bitter taste, side effects and endocrine disruption at higher doses limit its use [7,8,9]. The genotoxic and potentially adverse effects of genistein (apoptosis, inhibition of cell growth, topoisomerase inhibition, DNA damage) have been reported in vitro, as well as in experimental animals [10,11]. Poorly soluble flavonoids must be dissolved in order to be absorbed in the intestine, so various strategies are used to improve oral formulations (structural transformation, chemical modification (pro-drug-type glycosylation, metabolic coupling, prenylation), the addition of absorption enhancers, and, finally, formulation and technological processing (e.g., nanotechnology)) [12,13]. A number of nanotechnology formulations have been described to improve the biological activity of genistein, such as wet nanomilling and nanosuspension formation [14,15], the use of nanobubbles and lipid-based nanoemulsions to serve as carriers for genistein [16,17,18]. Advances in nanotechnology have made it possible to increase bioavailability, improve biological half-life and further the possibility of the targeted delivery of substances to the right site, thus enhancing the therapeutic potential of polyphenolic compounds [19,20,21,22]. On the other hand, modifying the size of a substance to the nanoscale dramatically changes the properties of the particles, primarily due to the increased surface area-to-volume ratio and the aggregation of nanoparticles that occurs, which further modifies their biological response [23]. Thus, it is not possible to assess the safety of nanocompounds based on the toxicological and ecotoxicological profile of the starting material at the macroscale. Ideally, the biological evaluation of nanoparticles should be verified on a case-by-case basis, taking into account particle size, organ/tissue and physiological state, and health/disease.

Genistein in its classical form has physicochemical properties similar to 17β-estradiol (E2), activating, in particular, the estrogen receptor Erβ, but with lower affinity than E2 [24]. Studies using human estrogen receptor (ER+) MCF-7 breast cancer cells indicate that physiological doses of E2 or genistein stimulate the growth of these cells in vitro and in vivo in raceless nude mice [25]. Therefore, estrogenic compounds, hormone replacement therapy and isoflavone supplements and soy products are generally not recommended in breast cancer patients [25]. It is also worth noting that other studies have shown that the in vitro treatment of estrogen-dependent and independent breast cancer cells with genistein at high doses inhibited their growth and stimulated it when genistein was administered at lower concentrations [26,27].

Genistein is also known to affect some physiological functions and kidney diseases [28,29]. Genistein exerts protective effects on normal cells by inhibiting oxidative stress, inflammatory reactions, and apoptosis. Genistein has beneficial effects on various kidney diseases by reducing symptoms, delaying disease progression and improving prognosis. In CDK (chronic kidney disease), as a result of calcium-phosphate disturbances, there is increased calcification in the cardiovascular system, as well as the formation of ecotopic, so-called metastatic calcifications in soft tissues [29]. These calcifications are in the form of amorphous calcium deposits in the tissue, located most often around large joints, as well as in parenchymal organs [30]. Genistein can regulate calcium uptake to maintain Ca^2+^ and phosphate balance and reduce vasodilation to stimulate diuresis [31]. In general, patients with chronic kidney disease require increased parathyroid work to maintain similar levels of bone turnover as those with normal kidney function. For example, dialysis patients have PTH levels two to three times higher than the standard amounts required during normal kidney function [32].

An increase in serum PTH (parathyroid hormone) levels in chronic kidney disease secondary to secondary hyperparathyroidism is an unavoidable complication accompanying end-stage renal disease due to the prolonged decline in serum Ca^2+^ and 1,25(OH) 2 D (1,25-dihydroxyvitamin D3) concentrations due to renal failure [33]. Secondary hyperparathyroidism includes an increase in PTH expression, synthesis, and secretion and excessive proliferation of parathyroid cells, resulting in parathyroid hypertrophy. Guo Y et al. [33] found that genistein can inhibit connective tissue growth factor expression and slow down the process of epithelial-to-mesenchymal transition, thus showing therapeutic potential against renal transdifferentiation and fibrogenesis in PTH-induced end-stage renal disease.

Our work focuses on ionic (especially calcium) imbalances, which can also lead to bone pathologies or other dysfunctions in the body as a consequence. We analyze the effect of the supplementation of various forms of genistein (nano, micro and classical) at a low dose (0.2 mg/kg, i.p.) on the content of 14 minerals in the kidneys of young rats under conditions of mammary gland tumorigenesis. Thus, the main purpose of this study was to try to determine the effect of selected forms of genistein on changes in the elemental composition of kidney tissue.

## 2. Materials and Methods

Genistein micro- and nanoforms were obtained by conventional milling and homogenization to reduce the size of particles. The preparation of genistein (micro- and nanoparticles) and parameters for evaluating the average particle size and zeta potential of the particles were described in our previous work [1].

### 2.1. Laboratory Animals

Sprague Dawley rats (30-day-old females; *n* = 32) were obtained from the Animal Science Laboratory of the Department of General and Experimental Pathology, Medical University of Warsaw. The Ethical Committee (Code 645/2018) approved the research protocol. Rats were fed a standard Labofeed H diet (standard diet: Labofeed H, Żurawia 19, 89-240 Kcynia, Poland; containing: 22.0% protein, 4.0% fat, 30.0% starch, 5.0% fiber, 6.5% minerals) and water ad libitum. The animals were kept in a controlled room at 22 °C with a 12 h light–dark cycle. The experimental study lasted 100 days. The animals adapted to the new conditions for 10 days, and then they were randomly divided into groups with an average of 8 rats.

Animals were divided into 4 study groups: control animals, who were not supplemented and received only a standard diet (instead of supplementation, they received 0.4 mL of water by gavage); animals supplemented by gavage with nanogenistein (0.1 mg/mL, i.e., 0.2 mg/kg.bw) (92 ± 41 nm); animals supplemented with microgenistein (0.1 mg/mL, i.e., 0.2 mg/kg.bw) (587 ± 83 nm); and animals supplemented with macrogenistein (normal, classic) (0.1 mg/mL, i.e., 0.2 mg/kg.bw). Supplementation was carried out from day 40 to week 20 of life (0.4 mL of aqueous suspension by gavage). Doses of polyphenols were selected while taking into account the average daily consumption by humans (calculated based on the body weight of rats). To induce mammary adenocarcinoma, rats were dosed twice (by gavage) with DMBA (7,12-dimethyl-1,2-benz(a)anthracene; Sigma-Aldrich, St. Louis, MO, USA) in rapeseed oil (at 60 days of age (80 mg/kg body weight); then, a dose of 40 mg/kg body weight was repeated at 90 days of age).

Interactive factors were eliminated by applying the same experimental procedure to all rats, i.e., age, experimental time, feed, housing conditions, tumor induction method, and supplementation method. The control group (standard diet—without supplementation) was given 0.4 mL of water instead of genistein to induce a similar level of stress to the animals in the control group.

During the experiment, rats were palpated to determine the time course of tumor development. The results obtained regarding tumor induction in the groups treated with 7,12-dimethylbenz[a]anthracene in relation to genistein supplementation—i.e., the rate of tumor appearance and the number of tumors—were presented in an earlier publication [1].

### 2.2. Elemental Level Testing

The solvents and reagents used in the analysis were of the highest commercially available purity. Ultrapure water (18 MΩ cm^−1^ resistivity) obtained from a Barnstead NANOpure Diamond UV system was used to prepare all standards and sample solutions. Samples were dissolved in 65% HNO_3_ and 37% HCl, Suprapur (Merck, Darmstadt, Germany). Multielement solutions of Ag, As, Al, B, Ba, Ca, Cr, Cu, Fe, Mg, Mn, Ni, Rb, Se, Sr, V and Zn, at a concentration of 10 mg/L (in 3% HNO_3_), were from Inorganic Ventures (Christiansburg, VA, USA). The stock standard solutions for Ca, Mg, K, Fe, Zn and Na (Merck, Germany) had a concentration of 1000 mg/L. The purity of argon (plasma gas) and helium (collision chamber gas) was over 99.999%.

### 2.3. Sampling

Elemental analysis of rat kidneys was performed. After collection, kidneys were frozen at −8 °C. Samples were thawed and then mineralized just before analysis. Kidney samples were placed in a hermetically sealed vessel containing 1 mL HCL and 4 mLHNO_3_. Samples were mineralized in a high-pressure laboratory microwave device (UltraWAVE T640, Milestone, Shelton, CT, USA (1500 W)). Heating was programmed in two stages. Initially, the temperature was gradually increased from 25 °C to 210 °C over 15 min, and then the temperature was maintained at 210 °C for 8 min. After mineralization, samples were diluted with water to a volume of 100 mL.

### 2.4. Instrumental Analysis

For lower-abundance elements (Ca, Zn, Fe, Cu, As, Se, Rb, Sr, Mo, B, Mn), a 7800 quadrupole ICP-MS (Agilent Technologies, Minato City, Tokyo, Japan) was used, containing an octopole collision cell. Measurements were made using nickel sample cones and a skimmer. For high-abundance elements—K, Mg, and Na—measurements were made using flame atomic absorption spectrometers—Solaar GF Zeeman and iCE3500 (Thermo Fisher Scientific, 168 Third Avenue, Waltham, MA, USA)—with single hollow cathode lamps using an air/acetylene flame. The following wavelengths were used: 766.5 nm, 285.2 nm, and 589.0 nm (for K, Mg and Na, respectively).

Before multielement analysis using ICP-MS and atomic absorption spectrometers, the analytical methods were checked using certificate material (water matrix reference material: EnviroMAT waste water, high (EU-H), catalog number 140-025-138, lot number S160225019 from SCP Science, Baie D’Urfé, QC, Canada).

### 2.5. Statistical Analysis

The Kruskal–Wallis test (followed by the post hoc Dunn test) was used for comparisons of quantitative variables in the four groups. Spearman’s correlation coefficient was used to assess the correlation between two quantitative variables. The significance level was set to 0.05. All the analyses were conducted in R software, version 4.3.1. (R Core Team (2023). R: A language and environment for statistical computing (R Foundation for Statistical Computing, Vienna, Austria. URL: https://www.R-project.org/) (accessed on 15 January 2024).

## 3. Results

### 3.1. Comparison of Kidney Weights of Rats Fed Different Diets (Standard and Supplemented with Macrogenistein, Microgenistein and Nanogenistein) in the Conditions of the Neoplastic Process

Comparing the ratio of kidney weight (g) to body weight (g), depending on the supplementation used, it was found that

- There were no differences in the body weight of the rats;
- In groups of rats supplemented with macrogenistein, microgenistein, and nanogenistein, there was an increase in relative kidney weights of 17%, 9% and 12%, respectively, compared to the standard group (Table 1).

### 3.2. Comparison of Mineral Content in Kidney Tissue of Rats Receiving Different Forms of Genistein—Macrogenistein, Microgenistein, and Nanogenistein—Without Supplementation (Standard Diet)

**Figure 1:**
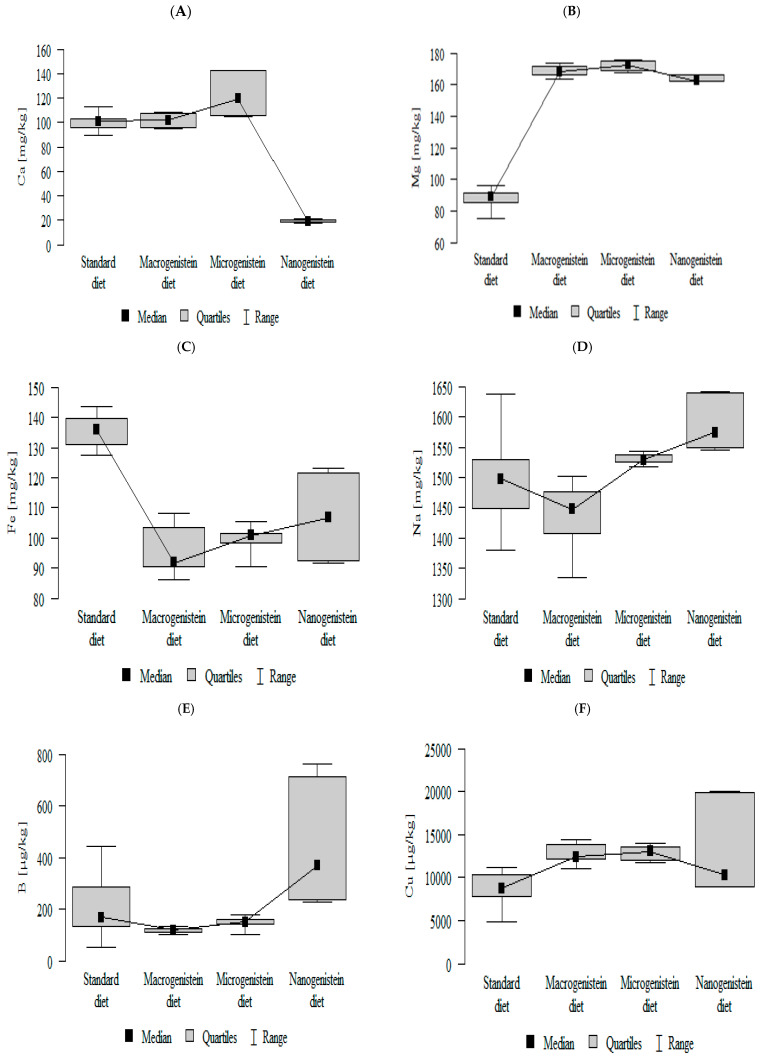

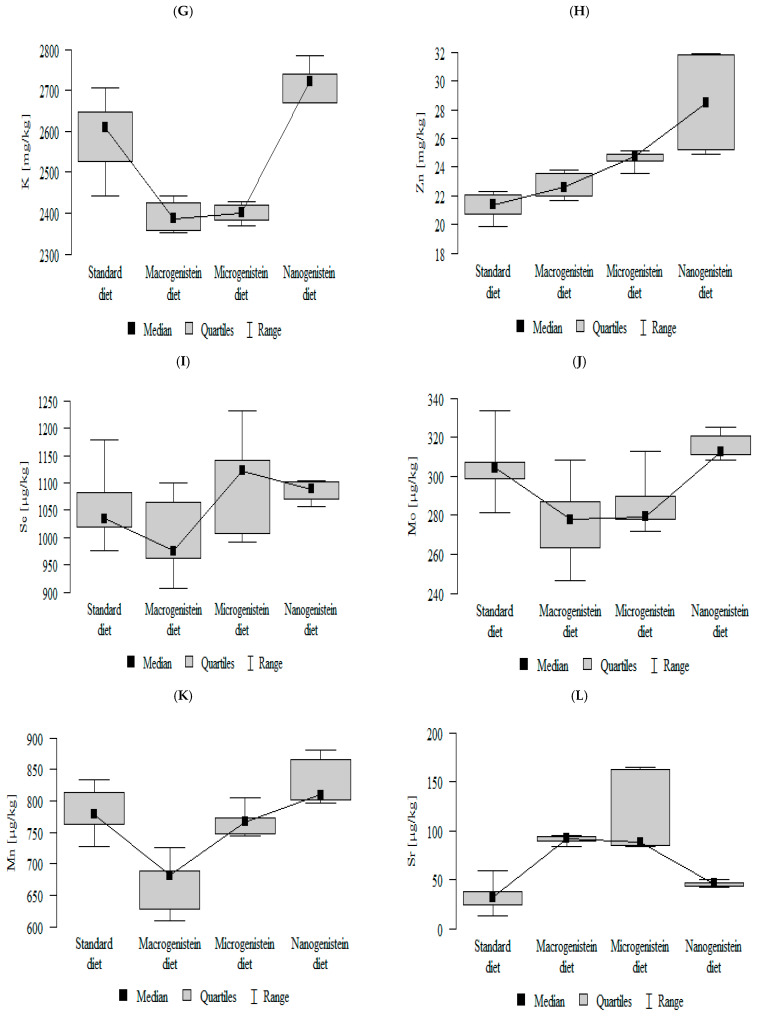

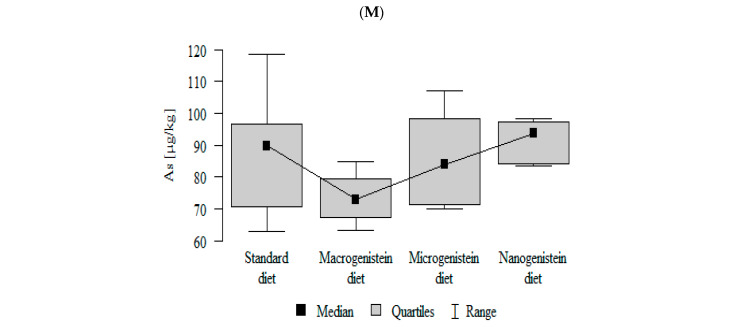
Minerals in the kidneys of rats with breast cancer supplemented with various forms of genistein: macrogenistein, microgenistein, nanogenistein, and no supplementation (standard diet) ((**A**)-calcium; (**B**)-magnesium; (**C**)-iron; (**D**)-sodium; (**E**)-boron; (**F**)-copper; (**G**)-potassium; (**H**)-zinc; (**I**)-selenium; (**J**)-molybdenum; (**K**)-manganese; (**L**)-strontium; (**M**)-arsenic).

The following results were obtained:

- The content of calcium was lower in the group with nanogenistein than in the other groups (Figure 1A; Table S1).
- The content of magnesium was lower in the control group (standard) than in the other groups (Figure 1B; Table S1).
- The content of iron was higher in the control group (standard) then in the other groups (Figure 1C; Table S1).
- The contents of sodium and barium were higher in the group with nanogenistein than in the other groups (Figure 1D,E; Table S1).
- The content of copper was lower in the control group (standard) than in the other groups (Figure 1F; Table S1).
- The content of potassium was higher in the control group (standard) and nanogenistein supplementation group than in the groups receiving micro- and macrogenistein (Figure 1G; Table S1).
- The content of zinc was higher in the groups supplemented with nano- or microgenistein than in the groups receiving macrogenistein and the control group (standard) (Figure 1H; Table S1).
- The content of selenium was higher in the groups supplemented with nano- or microgenistein than in the groups receiving macrogenistein (Figure 1I; Table S1).
- The contents of molybdenum and manganese were significantly higher in the group with nanogenistein than in the groups receiving micro- and macrogenistein. The level of molybdenum was higher in the control group compared to the group supplemented with macrogenistein. The level of manganese was higher in the control group (standard) and the group receiving microgenistein compared to the group receiving macrogenistein (Figure 1J,K; Table S1).
- The content of strontium was higher in the groups with macro- and microgenistein than in the groups receiving nanogenistein and the control group (standard) (Figure 1L; Table S1).
- The content of arsenic was higher in the group with nanogenistein than in the group receiving macrogenistein (Figure 1M; Table S1).

### 3.3. Analysis of Data on the Levels of Minerals in the Kidneys of Rats Receiving Different Forms of Genistein Under the Conditions of the Neoplastic Process (Figure 2, Figure 3, Figure 4, Figure 5 and Tables S2–S5 (Supplementary Materials))

**Figure 2:**
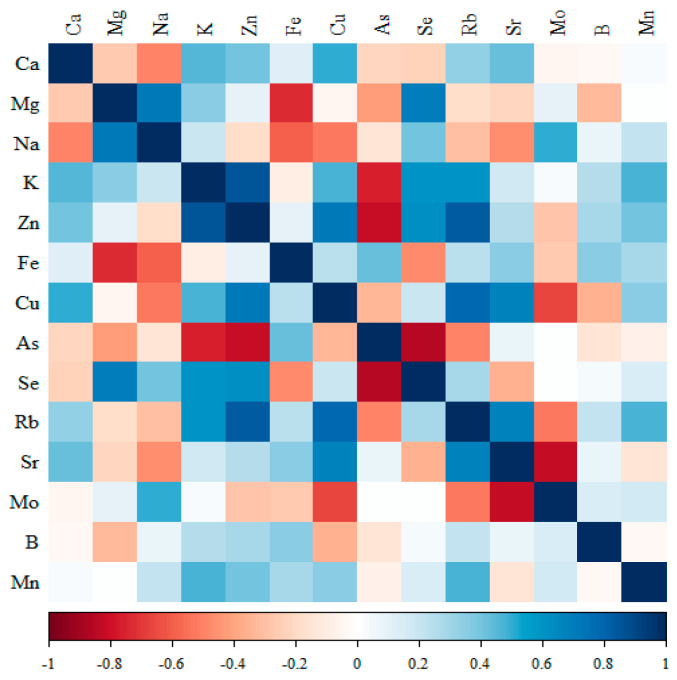
Heat map of correlations of 14 minerals in the kidneys of the control rats (standard diet) (blue indicates positive correlations and red indicates negative correlations).

**Figure 3:**
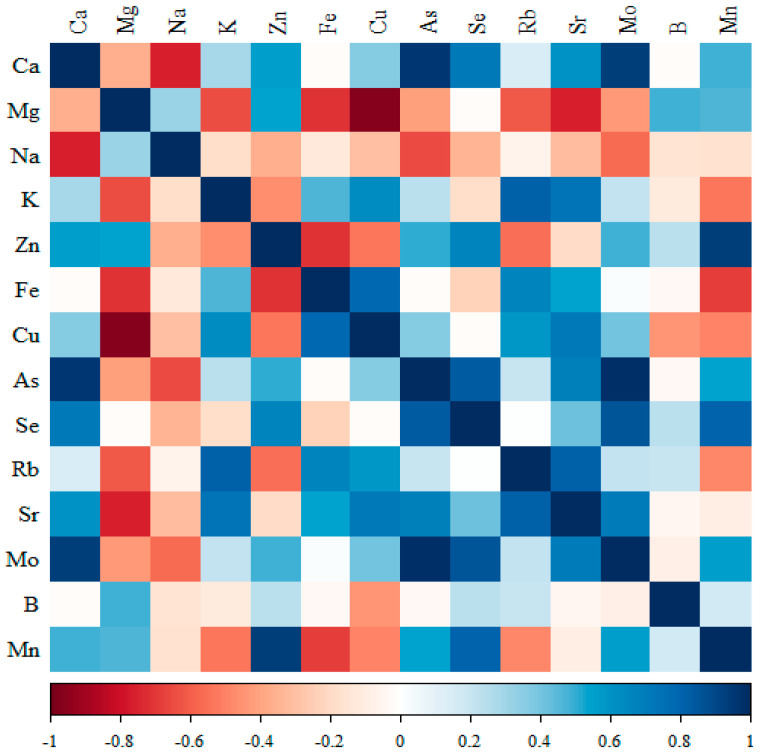
Heat map of correlations of 14 minerals in the kidneys of the group receiving macrogenistein (blue indicates positive correlations and red indicates negative correlations).

**Figure 4:**
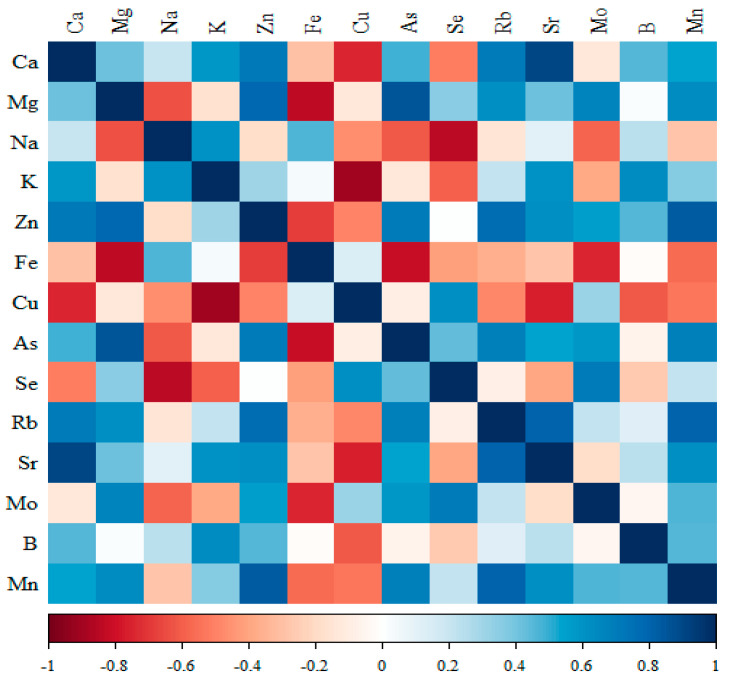
Heat map of correlations of 14 minerals in the kidneys of the group receiving microgenistein (blue indicates positive correlations and red indicates negative correlations).

**Figure 5:**
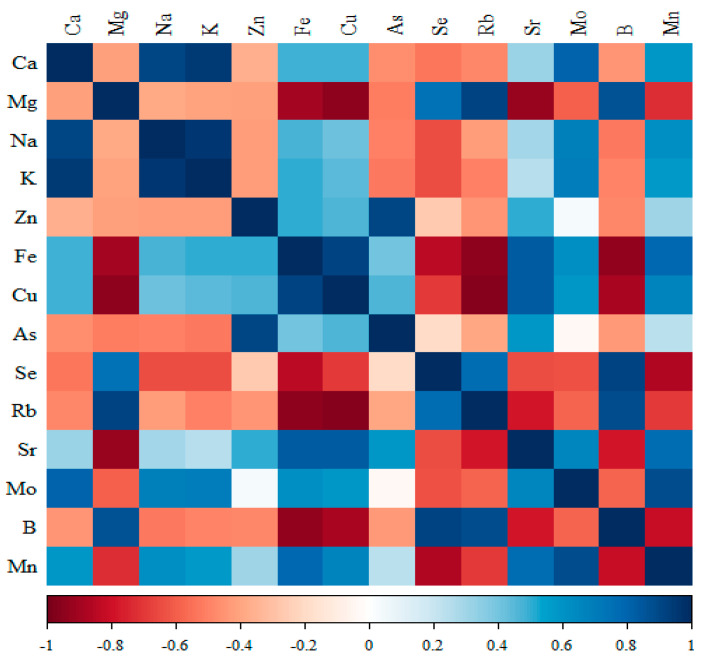
Heat map of correlations of 14 minerals in the kidneys of the group receiving the nanogenistein (blue indicates positive correlations and red indicates negative correlations).

The results of cross-correlations between elements, i.e., the type of expected changes, are shown in Figure 2, Figure 3, Figure 4 and Figure 5.

#### 3.3.1. Comparison of the Correlation of 14 Minerals in the Kidneys of the Control Group (Standard Diet)

Significant negative correlations were observed for Mg with Fe; As with K, Zn and Se; and Sr with Mo, and positive correlations were observed for K with Zn and Rb with Zn and Cu (Figure 2; Table S2).

#### 3.3.2. Comparison of the Correlation of 14 Minerals in the Kidneys of the Group Receiving Macrogenistein

Significant negative correlations were observed for Ca with Na; Mg with Fe, Cu and Sr; Na with Ca; and Zn with Fe, and positive correlations were observed for Ca with As, Se, and Mo; K with Rb and Sr; Zn with Mn; Cu with Fe and Sr; As with Ca, Se and Mo; Sr with Rb and Mo; and Mn with Se (Figure 3, Table S3).

#### 3.3.3. Comparison of the Correlation of 14 Minerals in the Kidneys of the Group Receiving Microgenistein

- Significant negative correlations were observed for Ca with Cu; Mg with Fe; Na with Se; K with Cu; Fe with As and Mo; and Cu with Sr.
- Positive correlations were observed for Ca with Zn, Rb and Sr; Mg with Zn and As; Zn with As, Rb and Mn; Se with Mo; Rb with Sr and Mn; and Mn with Rb (Figure 4, Table S4).

#### 3.3.4. Comparison of the Correlation of 14 Minerals in the Kidneys of the Group Receiving Nanogenistein

- Significant negative correlations were observed for Mg with Fe, Cu, Sr and Mn; Fe with Se, Rb and B; Cu with Se, Rb and B; Se with Mn; Rb with Sr and Mn; Sr with Rb and B; and B with Mn.
- Positive correlations were observed for Ca with Na, K and Mo; Mg with Se, Rb and B; Na with K and Mo; K with Mo; Zn with As; Fe with Cu, Sr and Mn; Cu with Sr; Se with Rb and B; Rb with B; Sr with Mn; and Mo with Mn (Figure 5, Table S5).

## 4. Discussion

The use of genistein at a low dose of 0.2 mg/kg.bw had no effect on the survival rate or final body weight of the animals. The animals grew throughout the experiment, and the same pattern of body weight changes was observed in each age group from the start of the study. The long-term 3-month administration of genistein independently to form under ongoing tumor conditions resulted in changes in kidney weight. The groups of rats supplemented with macrogenistein, microgenistein and nanogenistein had an enlargement of 17%, 9% and 12%, respectively, compared to the standard group. The observed renal hypertrophy and significant changes in mineral composition may be indicative of progressive renal damage and adverse effects, especially in the context of the giant calcifications in the rats’ femurs described in our previous article [34].

First of all, it is noteworthy that in the group of rats with nanogenistein supplementation, there was a strong, more than 80% loss in calcium levels in the kidneys of the rats compared to the other groups, and at the same time, there was a strong increase in the amount of magnesium in the kidneys in all the supplemented groups compared to the non-supplemented group by an average of 90%. There was also a decrease in iron content in the kidneys of the rats supplemented with various forms of genistein. In the work of Guo et al. [35], genistein was used at doses of 6 mg/kg and 20 mg/kg, and a reduction in the total relative kidney weight was obtained compared to mice without supplementation. The results of our study indicate that disorders of elemental homeostasis caused by bioactive compounds may involve not only typical, sometimes transient changes in blood serum but also changes in the state of internal organs, including the kidneys. Disorders of this organ, consequently, may result in osmotic and water–electrolyte changes in the whole body. Several studies have shown the effect of genistein on some physiological functions and kidney diseases (i.e., acute renal failure, kidney cancer). A reduction in symptoms, a delay in disease progression and an improvement in prognosis have been observed [28,36]. Thus, the prevailing consensus is that genistein has a beneficial effect on normal kidney cells through the mechanism of reducing the inflammatory response, apoptosis, and oxidative stress state and the regulation of blood pressure (renin), alongside the regulation of calcium and phosphate absorption and adequate diuresis. However, once renal cells are damaged, the protective effect of genistein diminishes, or it may even have an adverse effect [28,37].

We observed relatively insignificant renal hypertrophy (and perhaps glomerular hyperfiltration) with a maximum of several percent compared to the group of unsupplemented rats. This may be a sign of progressive renal damage, given the detected disruption of macronutrient homeostasis, particularly in the kidneys of nanogenistein-supplemented rats. In a number of papers, the use of the classical form of genistein has been shown to have a protective and balancing effect on calcium metabolism [28,31,38]. For example, the administration of 30 mg genistein/kg body weight/day for 3 weeks in male rats in an andropause model (orchidectomy) decreased the urinary Ca^2+^ content and increased the serum 25(OH) vitamin D content. It also showed decreased expression of FGFRs (fibroblast growth factor receptors) and PTH1R (parathyroid hormone 1 receptor) in the kidneys and increased expression of the gene and protein Klotho (transient activation of the TRPV5 Ca^2+^ channel in rat kidneys (transient receptor after potential receptor v-5))—which also contributed to calcium reabsorption [31]. This indicates that genistein is a potent biosurfactant with beneficial effects on the regulation of Ca^2+^ and phosphate homeostasis, especially in aging, when the balance of mineral metabolism is disturbed. However, in another study, treatment with genistein (10^−6^ M) abolished the effect of renal Ca^2+^ reabsorption, probably due to testosterone, which enhances Ca^2+^ transport by opening the T-type Ca^2+^ channel through mitogen-activated protein kinase (MEK)- and tyrosine kinase-dependent mechanisms in the rabbit kidney [39].

In our study, a form of nanogenistein likely impaired renal regulatory functions in the context of ion balance. The kidney is the main organ responsible for the elimination of metabolic products and potentially harmful substances, including nanoparticles [40]. Their removal is a multifaceted process involving glomerular filtration, tubular secretion, reflux resorption and the elimination of particles through urinary excretion. In vivo studies have shown that nanoparticles can have deleterious effects both at the tubular level, such as causing the degeneration of tubular epithelial cells and renal interstitial fibrosis, and at the glomerular level (causing glomerular edema, widening of Bowman’s space or mesangial cell hyperplasia). At any stage of excretion, the cytotoxic effects of various nanoparticles (illustrated by damage to the cell membrane, DNA, reduction in cell viability, induction of oxidative stress, or dysfunction of the cytoskeleton and mitochondria) may also occur on this organ, thus affecting its physiological functions [41], especially since the results of numerous studies indicate that nanoscale materials tend to move more strongly in the body compared to macroscale particles and can be deposited in organs and exhibit greater biological activity there [40,42,43]. Although they are of great concern from a toxicity perspective, little is known about the potential adverse effects of nanoparticles on these so-called secondary target organs [43,44]. Due to their function, the kidneys are therefore one of the most important secondary target organs for which nanoparticle toxicity should be carefully evaluated [40,45,46]. In our work, we analyzed the kidneys, as they are closely related to calcium homeostasis in the body (through PTH, vitamin D and blood Ca levels). Calcium ions and phosphate are key factors in maintaining the balance between bone formation and loss. When the calcium balance is disrupted, changes in bone composition leading to bone dysfunction will occur. In our previously published studies, we have shown that increased bone fragility takes place [34]. Thus, we can speak of an adverse effect of nanogenistein on calcium metabolism by an unknown indirect or direct mechanism. Bone structure is maintained by a dynamic balance between osteoblast-mediated bone formation and osteoclast-mediated bone resorption [47]. Genistein, due to its structural similarity to 17β-estradiol, can potentially prevent bone mass loss in postmenopausal women [47,48,49]. The main mechanism of action of genistein is the control of osteoblast metabolism through estrogen receptor (ER)-dependent pathways. However, a form of nanogenistein has led to excessive calcium deposition in bones by an unknown mechanism, possibly nanonephrotoxicity causing renal dysfunction. Healthy kidneys convert vitamin D obtained from food and sunlight exposure to its active form. Classic renal damage results in vitamin D deficiency in serum, inadequate calcium absorption and hypocalcemia [50]. In addition, in advanced disease, the kidneys are unable to remove phosphorus from serum. People with chronic kidney disease have an increased amount of phosphorus, which can bind to calcium and further reduce serum calcium levels [50,51]. However, renal failure can also lead to hypercalcemia as a result of secondary hyperparathyroidism [50]. Low levels of calcium in the blood activate the production of parathyroid hormone (PTH), which, among other things, increases calcium absorption by enhancing the formation of the active form of vitamin D and enhances the release of calcium from the bones. Eventually, the parathyroid glands can get out of control, resulting in persistently higher serum calcium levels [52,53]. Some work has shown that genistein lowers serum PTH levels and stimulates the expression of PTHR1 and sodium phosphate cotransporter 2a (NaPi 2a) in the kidneys and the expression of PTHR1 in the bones [38]. The kidneys, regulating the amount of calcium excreted, are a key checkpoint; in the glomeruli, about half of the plasma calcium (both in its ionic and complex forms) is filtered, while as much as 99% of the calcium is reabsorbed [54]. On average, an adult’s daily urine contains 200 mg of calcium. The TRPC5 and TRPC6 (transient receptor potential) channels play a key role in the renal reabsorption of calcium; mice lacking these proteins suffer from hypercalciuria and bone disease [55]. Efficient calcium transport in distal tubules is also enabled by the presence of calbindin D28K, the Na^+^/Ca^2+^ exchanger-1 (NCX1) protein and PMCA1a (plasma membrane calcium ATPase 1a) [54]. The strong expression of the Klotho protein in the kidneys and its regulation of several steps in calcium metabolism suggest its significant involvement in regulation. These mechanisms are not yet understood in detail, but studies in mice have shown that the absence of this protein results in high hyperphosphatemia. Similar abnormalities were detected in mice lacking fibroblast growth factor 23 (FGF 23) [54].

Our study took place under induced mammary gland cancer conditions. Tumor induction with DMBA was virtually 100%. It was confirmed histopathologically that these were grade 2 adenomas for the standard and genistein groups in the classical form and grade 3 for the supplementation of the micro and nanogenistein forms to the rats [1]. In the nanogenistein-supplemented animals, the first tumors appeared several weeks earlier than in the other groups, and by the end of the experiment, they had reached a larger size than in the standard group and classical genistein groups. It has long been known that phytoestrogens can have both estrogenic and anti-estrogenic effects, like SERMs (Selective Estrogen Receptor Modulators), depending on the concentration of circulating endogenous estrogens and estrogen receptors [56]. ERs (α and β) dimerize upon ligand binding, translocate to the cell nucleus and bind to estrogen response elements (EREs) in DNA sequences to facilitate site-specific gene transcription. These two transcriptional receptors have antagonistic functions in normal/healthy cell types, where both are expressed. ERα activation primarily induces cell growth (ERα is overactive in 50–80% of breast cancer cases). ERβ is a negative regulator of ERα and inhibits cell proliferation (presumably through the transcription of opposing genes rather than direct inhibition) [24]. ERβ is seen as a tumor suppressor and is indeed often mutated in advanced human cancers [57]. Genistein is a selective ERβ agonist, with about 20 times the affinity of ERα and a physiological concentration of only 8.4 nM providing 50% inhibition (IC50) [56].

The estrogenic effects of phytoestrogens were first observed as reproductive disorders in sheep [58]. The estrogenic effect of genistein can induce uterine hypertrophy, and the anti-estrogenic effect can reduce the uterine uptake of estradiol in animal models when this isoflavone is administered with estradiol. One determinant of estrogenic activity is SHBG (sex hormone binding globulin) levels. It has been found in in vitro studies that phytoestrogens, like estrogens, bind to estrogen receptors and increase the synthesis of SHBG by liver cells, but, like anti-estrogens, they inhibit aromatase activity and breast cancer cell proliferation. There have been many papers analyzing the effects of genistein, not only in the context of their action on estrogen receptors, but also their anti-inflammatory, antioxidant and cell cycle regulation activities, their effect on various signaling pathways, and their response to DNA damage [59,60,61]. At the molecular level, genistein has been shown to regulate proteins such as NF-kB, IL-1b, COX2, p53 and p21 [62,63,64,65,66,67].

However, the above considerations only apply to the classical form of genistein, while the nanogenistein form may generate different effects (beneficial and adverse), which are probably strongly dependent on both dose and particle size. Michael D. Kaytor et al. [68] evaluated the effects of nanogenistein on the efficacy of lung cancer radiotherapy in a mouse model using implanted A549 lung adenocarcinoma cells (NSCLCs). The researchers used a nanosuspension supply of genistein (containing 325 mg/mL synthetic genistein with a d(50) particle size distribution of less than 0.2 μm), administered by gavage at 200 mg/kg and 400 mg/kg daily before and after exposure to a single dose of radiation. Animals that received nanogenistein at the higher dose showed reduced tumor growth and improved normal lung histopathology compared to the radiation alone group. However, survival analysis showed that animals receiving nanogenistein alone at a higher dose had a shorter survival time compared to irradiated animals that were also given nanogenistein, although the authors did not consider this to be a direct cause of the treatment used but stressed the need for longer studies [68,69].

Another study also showed that the administration of nanogenistein at a dose of 200 mg/kg (BIO 300 formulation) inhibits the growth of prostate cancer tumors, alone or in combination with radiotherapy, in mouse models (Athymic Nude-Foxn1nu) with implanted PC3 and LNCaP prostate cancer cells [70]. The radioprotective effects of genistein on the bone marrow and spleen were also studied. Genistein was also administered as a wet-milled nanosuspension with a particle size of 200 nm at a dose of 150 mg/kg. The genistein nanosuspension was shown to inhibit the production of inflammatory factors in irradiated mouse bone marrow and spleen cells, which may contribute to the survival of hematopoietic progenitor cells [56,63]. However, it has been emphasized that a single intramuscular injection after irradiation is not effective in terms of increasing animal survival. In addition to protecting from radiation for acute radiation-induced hematopoietic damage, genistein alone or in combination with other compounds has also been shown to protect other organs, including the liver, testes and just the kidneys [71,72,73]. Kidney damage after radiation exposure and the administration of the genistein nanosuspension at a dose of 100 mg/kg after 24 h was assessed by the presence of tubular atrophy or mild, moderate or severe tubular damage [74]. In the kidneys, irradiation leads to the progressive deterioration of function associated with coexisting glomerular sclerosis and/or tubulointerstitial fibrosis or interstitial tubular damage. Morphological and physiological studies have shown that the renal tubular system is the site with the greatest radiation damage. The renal MDA levels in the γ-irradiation and 100 mg/kg nanogenistein (RT+G) groups were significantly reduced compared to the γ-irradiation-only (RT) group [73]. DNA repair is crucial for anti-radiation efficacy, and further studies are needed to determine whether genistein in vivo regulates ERβ-mediated DNA repair genes. The in vitro data presented here confirm that genistein, as a nanosuspension, had ~2000-fold selectivity in activating ERβ over Erα. However, caution should be exercised when considering potential pharmaceutical applications of genistein, as many reports on its biological activity have used high molar concentrations in cell cultures that are not relevant in vivo.

However, in our study, even the use of nanogenistein at a much lower dose—0.2 mg/kg. mc (92 ± 42 nm), a dose extrapolated from the average intake of isoflavone compounds—translated to very large changes in kidney mineral composition and perhaps function. Also, the impact of the ongoing cancer process on both kidney function and bone mineral status cannot be ruled out, if only through affecting the hormones and factors secreted by cancer cells, especially since there is an increased risk of cancer in patients with chronic kidney disease, and cancer patients are more likely to have chronic kidney disease. Some researchers [75,76] claim that acute kidney injury and chronic kidney disease can both cause and result in cancer. It is likely that renal dysfunction creates a peculiar inflammatory microenvironment and oxidative stress state, which may promote or exacerbate cancer. Many causes of kidney disease in cancer patients have been established/identified, including acute kidney injury, electrolyte imbalance and acid–base disorders [77]. Some causes of chronic kidney disease in cancer patients in addition to the use of chemotherapeutic drugs are nephropathies and various types of glomerulonephritis [75,76,77,78]. Interestingly, the most common type of renal dysfunction is due to nephrocalcinosis, a generalized increase in calcium content in the kidneys [79]. Renal calcification can occur at the molecular, microscopic or macroscopic level, leading to progressive kidney damage. Among the main causes of this are those associated with an increase in urinary calcium, oxalate and phosphate levels. Under these conditions, the concentration and supersaturation of urine lead to the precipitation of calcium crystals, which can take place inside the tubules or start in the renal interstitium. Renal calcification is a dangerous and sinister phenomenon and is often associated with a serious metabolic defect [79].

In our study, the opposite phenomenon occurred in terms of calcium, while there was a large, nearly 100% increase in magnesium in all groups and a 33% increase in zinc in the kidneys of rats supplemented with micro- and nanogenistein compared to the non-supplemented standard group. Renal work significantly influences the maintenance of serum magnesium homeostasis by regulating the excretion of this element in urine. The wide spectrum of the excretory capacity of the kidneys is evidenced by their ability to remove 70% of the magnesium ion load in the case of hypermagnesemia, and in the state of hypomagnesemia, they can reduce excretion to 0.5% [80]. The main factors influencing magnesium reabsorption include the plasma concentration of the element, the phosphate concentration (a decrease in phosphate concentration leads to hypomagnesemia) and hormones—parathormone (increases absorption in distal tubules) and calcitonin [81]. Magnesium has a protective function against calcification, which has been proven in vitro [82]. In the course of chronic renal failure, hyperphosphatemia and hypercalcemia are factors that accelerate fibrosis and thus disease progression; however, further studies are required to conclusively determine the beneficial effects of magnesium in chronic kidney disease [83,84]. Magnesium competes with calcium and reduces its concentration, which leads to the relaxation of smooth muscle cells, and then there is a dilation of blood vessels, which can have a positive effect on hypoxic conditions [85]. Hypermagnesemia is quite rare, and it occurs mainly in patients with acute or chronic kidney disease, and with a high intake of magnesium in the form of supplements or magnesium-containing laxatives or antacids, hypothyroidism and adrenal insufficiency can also contribute to this condition [80]. The kidneys play a key role in maintaining normal Mg concentrations; when the glomerular filtration rate decreases, the kidneys’ ability to excrete Mg decreases accordingly [86,87]. However, in advanced chronic kidney disease, the fraction of filtered Mg excreted increases due to impaired tubular reabsorption [87,88,89]. On the other hand, hypermagnesemia inhibits parathyroid hormone secretion [89]. A study by Contiguglia et al. [90] found that patients with chronic renal failure have increased magnesium concentrations throughout the body (i.e., heart muscle, lungs, red blood cells and skin), with bones being the main reservoir of magnesium. In our study on changes in bone mineral composition [34], there was a significant reduction in this element in the genistein-supplemented groups, especially for the micro- and nanoforms, compared to the non-supplemented group. Acute kidney damage can occur during ischemia or due to increases in nephrotoxic substances; renal tubular dysfunction and disruption of water–electrolyte homeostasis occur in this context [91]. It cannot be ruled out that the supply of nanogenistein in the dose and size used caused electrolyte disturbances.

On the other hand, the state of reduced iron and increased zinc in the kidneys under the experimental conditions is very difficult to explain. In most works, the dominant view is that excess iron plays a negative role and an increase in free radicals causes damage to important molecules and tissues in the kidneys (the result can be proteinuria in glomerular disease or progressive diabetic nephropathy, among others) [92,93]. It has been found that the amount of iron deposition in kidney tissue can be both a cause and a progressive factor in existing chronic kidney disease and can be a sensitive indicator of kidney damage [92,93,94]. Most studies involve in vitro animal models or cell cultures, but they are strong enough to postulate the investigation of various antioxidants and/or iron chelators for therapy in renal failure [93,95]. The metabolism of the kidneys is very intensive; the large number of mitochondria in the cells of this organ leads to high levels of iron, and iron proteins and sulfur are important for the functioning of the respiratory chain [95,96].

Iron undergoes filtration in the glomeruli and is then reabsorbed in the renal tubules. This includes both transferrin-bound and non-transferrin-bound iron (the transferrin-bound iron fractions (TBI and NTBI) are absorbed—a process made possible by the presence of transferrin receptors on the surface of proximal tubules and the process of endocytosis mediated by megalin and cubilin receptors [97]). During reabsorption in the loop of Henle, iron competes with other divalent metals such as copper. The kidneys have the highest expression of IRP1 (iron regulatory protein 1) among all other organs; this protein controls the cytosolic iron content and regulates the expression of components of ferritin, transferrin and other iron proteins. IRP1 regulates iron metabolism in the kidneys [98].

On the other hand, anemia is a common complication in patients with chronic kidney disease (CKD), as a result of the shortened survival of red blood cells caused by uremia and the relative deficiency of erythropoietin due to insufficient production by the kidneys [95,99,100]. It has also been observed that during the course of chronic diseases such as immune disorders, recurrent infections or cancer, anemia of chronic disease (ACD) also occurs [101].

The approximately 23% decrease in the renal iron content in the genistein-supplemented rats found in our study, regardless of form, compared to the standard group may be indicative of impaired renal iron homeostasis, perhaps related to excess zinc and copper or/and tumor induction and increased tumor demand for this element.

In the case of zinc, we showed an approximately 30% increase in its content in the kidneys of rats supplemented with nanogenistein and microgeneistein in particular, compared to the standard group, and an almost 50% increase in the amount of copper in the kidneys of rats supplemented with various forms of genistein compared to the non-supplemented group. It should be noted, however, that most studies on zinc in a nephrological context are concerned with zinc deficiency states associated with kidney disease and comorbidities [102,103,104].

In the course of chronic kidney disease, especially in the advanced stage of the disease, there are reduced plasma zinc concentrations [105]. Zinc is involved in modulating the pro-inflammatory response by acting on nuclear factor Kappa B (NF-κB), a transcription factor that is a major regulator of pro-inflammatory responses. It is also involved in controlling oxidative stress and regulating inflammatory cytokines. Zinc therefore has a complex function during the immune response, and its homeostasis is crucial for maintaining the proper functioning of the immune system [106]. Under conditions of oxidative kidney damage induced by lead acetate exposure, it was found that zinc administration showed a nephroprotective effect; in addition, it also inhibited the excessive breakdown of vitamin E, presumably as a result of reducing the formation of reactive oxygen species and improved renal parameters such as ALP (alkaline phosphatase, one of the markers of acute kidney injury) and LDH (lactate dehydrogenase, a non-specific parameter that is observed to increase in acute kidney injury) [107]. Thus, taking into account the clinical studies depicting the reciprocal correlations between zinc and chronic kidney disease, as well as numerous animal studies, it can be inferred that zinc deficiency plays an important role in the progression of kidney disease [104]. Meanwhile, in our study, zinc levels increased in the kidney tissue of rats supplemented with a miniaturized form of genistein compared to the non-supplemented group, as did copper content.

The most commonly cited disease associated with abnormal copper metabolism is Menkes disease, resulting from a mutation of ATP7A, which is necessary for the coding of Cu-ATPase, required for copper absorption in the intestines and its subsequent passage into circulation. This mutation causes copper deficiency in all tissues except the epithelium of the intestinal and renal tubules. The main treatment for patients with Menkes syndrome is the subcutaneous injection of copper ions. One negative aspect is accumulation and severe damage to the kidneys, including renal tubular necrosis [108]

Lenartowicz et al. [108] claim that excess copper can impair Na(+)/K(+) ATPase activity in the renal tubules of male mice with a mosaic mutation (an animal model of Menkes disease). The contents of Mg, P and Cl in the kidneys of mouse mutants are also altered, especially after copper administration. The approximately 50% increase in copper content shown in our study in the kidneys of rats receiving all forms of genistein compared to a diet without supplementation may suggest that copper has a toxic effect (renal tubular necrosis and abnormal proliferation of renal tubule cells). Cu is believed to play a role in renal function due to its active redox nature [109]. Cu has been found to be involved in the physiological processes of oxidative stress and inflammation, which play an important role in the pathogenesis of PChN [110]. In vivo studies have observed that overexposure to Cu can induce oxidative stress, which causes autophagy and apoptosis in rat kidneys [111,112,113].

Can the supply of genistein be combined with copper concentration? It has been proven that regular genistein is cytotoxic to cancer cells, but this is not the case for normal cells [114]. Such preferential cytotoxicity to cancer cells can be explained by the fact that serum, tissue and intracellular copper levels are significantly elevated in various malignancies [115,116]. Copper transporters are overexpressed in malignant cells, which helps in the uptake and accumulation of excess copper [117]. Because cancer cells contain elevated levels of copper, they may be more susceptible to redox reactions, generating excess ROS. Most cancer cells are deficient in antioxidant enzymes compared to normal cells. In cancer cells, ROS levels can exceed the cells’ antioxidant capacity, leading to irreversible damage and apoptosis [118]. One mechanism of genistein-induced cytotoxicity against cancer cells is the mobilization of endogenous copper and the resulting pro-oxidant effect, as well as consequent cell death. Interestingly, it was previously shown that normal MCF10A breast epithelial cells do not contain detectable amounts of copper [119], which could explain their resistance to genistein. Nanoparticles of genistein, presumably depending on their size (miniaturization size), may interact completely differently. Also, in the example of copper, the chemical form modifies toxicity. The work of Chen et al. [120] linked copper nanoparticles and microparticles to the degree of toxicity. Copper nanoparticles showed high absorption and migration rates and high ionization potential. In a mouse study, they caused glomerulonephritis and the degeneration and necrosis of renal tubules; such effects were not observed in the copper microparticle group. The toxicity of nanocopper was also shown to be sex-dependent, with males presenting with more severe physiological and biochemical disorders. The authors associate the harmfulness of copper nanoparticles with the overproduction of bicarbonate ions, which cannot be excreted by faulty kidneys, consequently leading to metabolic alkalosis [120].

Boron was another element whose increased concentration was noted in this study. There was a more than 100% increase in boron concentration in the kidneys of nanogenistein-supplemented rats relative to all other groups of rats. Since the kidneys are the main route of boron excretion, the accumulation of this element in patients with renal failure is likely [121]. The accumulation of boron in the kidneys can damage the renal tubules, and at higher doses of 320–640 mg/L, cell apoptosis can occur [122]. In one study [123], rats were orally exposed to boric acid (100–275–400 mg/kg/day). At the end of the experiment (10th, 30th and 45th day), kidney weight, the boron concentration in the kidneys and histopathological changes were determined. In the experimental group, a significant accumulation of boron in renal tissue was observed, with a significant decrease in boron concentration on day 45 compared to day 30. Histopathological degenerative changes were observed, especially in proximal tubule cells, which were dose- and time-dependent. Subacute exposure to boric acid caused dose-dependent histopathological changes in renal tissue [123]. There was also a significant reduction in Nrf2 expression, with the HO^−1^ concentration in the groups of animals with BA supplementation being higher than 160 mg/L. In summary, the data from this study indicate that low doses of boron (especially 80 mg/L BA) had beneficial effects on the kidneys of ostrich chicks by improving antioxidant capacity and enzyme activity and inhibiting cell apoptosis; however, high levels of boron (160, 320, 640 mg/L BA) had adverse effects. This study shows, for the first time, the underlying mechanism of boron’s effect on ostrich chicks’ kidneys, which is related to the regulation of the Nrf2 pathway and cell apoptosis. Thus, at low doses of boron, the antioxidant capacity improved. However, as the amount of BA increases, the negative effects may override the antioxidant defense system, thus causing detrimental effects on its development [123].

In our study, we also observed a slight increase in sodium, potassium and selenium contents, with a maximum of several percent, in the kidneys of the rats supplemented with nanogenistein relative to the other study groups. It is thought that the more patients with chronic kidney disease show a higher ratio of sodium and potassium in their 24 h urine, the more rapidly renal function may deteriorate [124]. Sodium intake increases the intravascular blood volume, raising blood pressure and thus increasing glomerular pressure. The overall effect is a deterioration of kidney function. Potassium has the opposite effect to sodium and therefore increases its excretion. Reducing the intravascular volume and decreasing the vascular resistance lower blood pressure, which in turn lowers glomerular pressure and stabilizes kidney function. Therefore, potassium intake should have a positive effect on renal function. However, as GFR decreases, potassium excretion becomes less effective and the blood potassium concentration increases [125,126]. In our study, the disturbances of these two very important cations for electrolyte balance were relatively small. Also, the Na/K quotient was similar in all study groups.

Decreased levels of selenium, and thus possibly glutathione peroxidase, are noted in chronic renal failure, which may lead to susceptibility to oxidative stress [127,128]. In whole blood, plasma and red blood cells, a proportion of reduced selenium concentrations during disease progression has been observed [129]. Patients with chronic kidney disease, especially those on hemodialysis, are more susceptible to oxidative stress, and disturbances in homeostasis can lead to numerous complications [130]. Recent studies have confirmed a positive correlation between plasma selenium levels and eGFR in a dose-dependent manner in a group of patients with cadmium- and lead-induced chronic renal failure [131]. In the present study, there was an average 9% increase in selenium regarding its content in the kidneys of rats receiving nanogenistein and microgenistein compared to the group receiving the classical form of genistein (macrogenistein).

Animal studies over the past decade have shown that some nanoparticles can reach important organs such as the heart, brain and kidneys, cause biochemical damage and contribute to the development of disease [132,133,134,135,136,137,138,139,140]. An evaluation of the potential toxicity of genistein nanoparticles at the size and dose used, i.e., 0.2 mg/kg.bw due to the detected mineral disturbances in the kidneys and bones, seems necessary to ensure their safe use.

## 5. Conclusions

On the basis of this study, it was shown that the supplementation of the animals’ diet with nanogenistein, microgenistein and macrogenistein had an effect on the differences in the concentrations of the elements in question and their mutual correlations in the kidneys of rats with breast cancer. Particularly negative effects of nanogenistein administration on the amount of calcium and iron were found in vivo, evidencing increased distribution or excretion of these elements from the body, as well as an increase in the number of elements, especially the concentrations of magnesium, sodium, zinc, boron and copper, compared to the non-supplemented (standard) group. These data indicate that nanoparticles may adversely affect renal health, and further studies are needed to clarify the mechanisms of these changes and perhaps their nephrotoxicity.

## Figures and Tables

**Table 1:** Body mass of rats and percentage of femur mass in final body mass (%).

Groups	Final Body Weight ± SD (g)	Kidney Weight ± SD (g)	Ratio ± SD (%)	*p*
Standard	233.3 ± 17.3	1.636 ± 0.197	0.7 ± 0.06	
Macrogenistein	214.5 ± 7.1	1.750 ± 0.104	0.82 ^a^ ±0.05	*p* ≤ 0.001
Microgenistein	225.9 ± 13.9	1.708 ± 0.082	0.76 ^c^ ± 0.04	*p* ≤ 0.05
Nanogenistein	221.1 ± 10.4	1.745 ± 0.145	0.79 ^b^ ± 0.07	*p* ≤ 0.02

Results are presented as means ± SEM; statistically significant results are ^a^—*p* ≤ 0.001; ^b^—*p* ≤ 0.02; ^c^—*p* ≤ 0.05; and relative to the standard group.

## Data Availability

The data presented in this study are available on request from the corresponding author.

## Supplementary Materials

The following supporting information can be downloaded at https://www.mdpi.com/article/10.3390/nu17071184/s1, Table S1: Mineral composition of rat kidneys; Table S2: Comparison of the correlation of 14 elements in the kidneys of rats receiving a standard (non-supplemented) diet; Table S3: Comparison of the correlation of 14 elements in the kidneys of rats receiving the macrogenistein diet; Table S4: Comparison of the correlation of 14 elements in the kidneys of rats receiving the microgenistein diet; Table S5: Comparison of the correlation of 14 elements in the kidneys of rats receiving the nanogenistein diet.
