# Supplementary material for: The Genistein Supply and Elemental Composition of Rat Kidneys in an Induced Breast Cancer Model
Source: Nutrients. 2025 Mar 28;17(7):1184. doi: 10.3390/nu17071184 (PMC11990330; doi:10.3390/nu17071184)
Supplement: Supplementary file 1 [file nutrients-17-01184-s001.zip › nutrients-3549111-supplementary.pdf]

Table S1. Mineral composition of rat kidney.

Parameter	Diet	N	Mean	SD	Median	Min	Max	Q1	Q3	P
Ca [mg/kg]	Standard diet	8	100.41	7.15	100.99	89.36	113.35	96.31	103.29	p<0.001 *
	Macrogenistein diet	9	101.74	5.59	101.79	94.85	108.48	95.51	107.96	micro,macro,stand>nano
	Microgenistein diet	9	122.35	16.41	119.47	104.82	142.81	105.40	142.52	
	Nanogenistein diet	9	19.47	1.04	19.09	18.04	20.92	18.67	20.18	
Mg [mg/kg]	Standard diet	8	87.08	7.22	88.91	75.24	95.88	85.19	91.30	p<0.001 *
	Macrogenistein diet	9	168.77	3.30	168.11	163.82	173.62	166.47	171.88	micro,macro,nano>stand
	Microgenistein diet	9	172.21	3.23	172.66	167.59	175.76	169.02	175.35	
	Nanogenistein diet	9	163.69	1.81	162.59	162.20	166.22	162.48	166.04	
Na [mg/kg]	Standard diet	8	1497.63	79.34	1497.14	1379.53	1637.15	1449.74	1529.47	p<0.001 *
	Macrogenistein diet	9	1435.64	53.70	1447.04	1333.84	1502.64	1407.37	1475.92	nano>micro,stand,macro micro>macro
	Microgenistein diet	9	1530.78	9.43	1528.79	1517.10	1542.92	1526.40	1537.93	
	Nanogenistein diet	9	1587.00	41.61	1574.72	1544.83	1641.57	1549.76	1638.82	
K [mg/kg]	Standard diet	8	2585.94	96.23	2608.38	2441.93	2707.75	2526.91	2647.60	p<0.001 *
	Macrogenistein diet	9	2389.28	36.25	2386.68	2353.01	2441.00	2357.82	2425.65	nano,stand>micro,macro
	Microgenistein diet	9	2399.90	21.72	2401.40	2368.01	2429.21	2383.98	2419.22	
	Nanogenistein diet	9	2720.37	44.27	2720.91	2669.34	2785.13	2670.43	2739.91	
Zn [mg/kg]	Standard diet	8	21.28	0.88	21.38	19.82	22.32	20.75	22.04	p<0.001 *
	Macrogenistein diet	9	22.74	0.82	22.60	21.64	23.82	22.01	23.57	nano,micro>macro,stand
	Microgenistein diet	9	24.58	0.48	24.75	23.56	25.10	24.41	24.86	
	Nanogenistein diet	9	28.43	2.95	28.46	24.87	31.91	25.24	31.82	
Fe [mg/kg]	Standard diet	8	135.40	5.71	135.88	127.53	143.49	131.08	139.53	p<0.001 *
	Macrogenistein diet	9	96.10	7.98	91.78	86.07	108.00	90.61	103.39	stand>nano,micro,macro
	Microgenistein diet	9	99.36	4.84	100.71	90.41	105.35	98.48	101.36	
	Nanogenistein diet	9	106.95	13.14	106.69	91.62	123.00	92.26	121.48	
Cu [µg/kg]	Standard diet	8	8728.60	2006.39	8800.98	4933.68	11246.54	7880.71	10297.01	p=0.002 *
	Macrogenistein diet	9	12788.29	1110.05	12429.81	11089.15	14465.54	12139.73	13811.34	micro,macro,nano>stand
	Microgenistein diet	9	12909.74	823.94	13030.85	11757.33	14060.78	11992.68	13538.56	

Parameter	Diet	N	Mean	SD	Median	Min	Max	Q1	Q3	P
	Nanogenistein diet	9	13078.61	5192.11	10299.46	8972.18	20040.37	9003.21	19868.59	
As [µg/kg]	Standard diet	8	87.70	19.35	89.76	63.01	118.64	70.67	96.76	p=0.033 *
	Macrogenistein diet	9	73.54	7.17	72.96	63.37	84.92	67.36	79.40	nano>macro
	Microgenistein diet	9	85.37	13.98	83.89	69.96	107.09	71.32	98.23	
	Nanogenistein diet	9	91.69	6.42	93.74	83.42	98.23	84.03	97.40	
Se [µg/kg]	Standard diet	8	1050.14	65.71	1034.31	976.71	1178.25	1019.20	1081.51	p=0.008 *
	Macrogenistein diet	9	996.40	65.88	975.97	906.54	1100.45	962.00	1065.45	micro,nano>macro
	Microgenistein diet	9	1100.00	81.83	1122.19	992.15	1231.54	1007.51	1141.28	
	Nanogenistein diet	9	1083.20	19.40	1088.85	1056.89	1104.76	1069.73	1101.20	
Rb [µg/kg]	Standard diet	8	3825.14	241.29	3883.75	3446.72	4133.77	3753.77	3954.31	p=0.255
	Macrogenistein diet	9	3577.24	288.80	3460.72	3245.59	4001.86	3357.91	3891.41	
	Microgenistein diet	9	3642.78	306.13	3768.36	3240.54	3929.21	3266.28	3898.26	
	Nanogenistein diet	9	3952.76	434.42	3768.53	3551.88	4542.09	3617.08	4483.11	
Sr [µg/kg]	Standard diet	7	32.88	14.71	32.00	13.42	59.59	24.62	37.96	p<0.001 *
	Macrogenistein diet	9	91.46	3.78	92.03	83.69	94.93	89.01	94.59	macro,micro>nano,stand
	Microgenistein diet	9	112.44	38.97	87.85	83.73	165.45	84.90	162.66	
	Nanogenistein diet	9	45.75	2.39	46.12	41.78	49.68	43.75	46.72	
Mo [µg/kg]	Standard diet	8	304.47	14.79	304.39	281.62	333.66	298.97	307.48	p<0.001 *
	Macrogenistein diet	9	276.70	18.30	277.81	246.73	308.46	263.35	287.15	nano>micro,macro<br>stand>macro
	Microgenistein diet	9	284.75	13.31	279.38	271.83	313.02	277.98	289.62	
	Nanogenistein diet	9	315.28	6.04	312.49	308.25	325.40	311.17	320.96	
B [µg/kg]	Standard diet	7	213.77	132.52	167.01	52.78	442.35	131.55	285.57	p<0.001 *
	Macrogenistein diet	9	117.14	10.12	117.80	100.33	132.83	110.85	123.74	nano>stand,micro,macro<br>stand>macro
	Microgenistein diet	9	146.67	24.67	150.35	102.62	179.26	143.61	161.29	
	Nanogenistein diet	9	449.83	231.07	368.57	226.43	763.13	237.69	715.39	
Mn [µg/kg]	Standard diet	8	783.96	36.00	779.01	727.69	833.49	763.08	813.60	p<0.001 *
	Macrogenistein diet	9	663.41	40.72	681.78	609.99	726.10	628.43	688.87	nano>micro,macro<br>stand,micro>macro
	Microgenistein diet	9	764.84	19.44	766.40	745.04	805.25	747.88	772.77	

Parameter	Diet	N	Mean	SD	Median	Min	Max	Q1	Q3	P
	Nanogenistein diet	9	828.85	35.82	810.05	796.35	881.63	801.92	866.48	

Differences were considered statistically significant when $p < 0.05$.

Table S2. Comparison of the correlation of 14 elements in the kidney of rats receiving a standard (non-supplemented) diet.

	Ca	Mg	Na	K	Zn	Fe	Cu	As	Se	Rb	Sr	Mo	B	Mn
**Ca**	---	r=-0.262, p=0.536	r=-0.5, p=0.216	r=0.452, p=0.267	r=0.405, p=0.327	r=0.119, p=0.793	r=0.5, p=0.216	r=-0.214, p=0.619	r=-0.238, p=0.582	r=0.333, p=0.428	r=0.429, p=0.354	r=-0.048, p=0.935	r=-0.036, p=0.963	r=0.024, p=0.977
**Mg**	r=-0.262, p=0.536	---	r=0.714, p=0.058	r=0.357, p=0.389	r=0.095, p=0.84	r=-0.738, p=0.046 *	r=-0.048, p=0.935	r=-0.429, p=0.299	r=0.69, p=0.069	r=-0.19, p=0.665	r=-0.214, p=0.662	r=0.095, p=0.84	r=-0.321, p=0.498	r=0, p=1
**Na**	r=-0.5, p=0.216	r=0.714, p=0.058	---	r=0.19, p=0.665	r=-0.19, p=0.665	r=-0.595, p=0.132	r=-0.524, p=0.197	r=-0.143, p=0.752	r=0.405, p=0.327	r=-0.31, p=0.462	r=-0.464, p=0.302	r=0.5, p=0.216	r=0.071, p=0.906	r=0.214, p=0.619
**K**	r=0.452, p=0.267	r=0.357, p=0.389	r=0.19, p=0.665	---	r=0.857, p=0.011 *	r=-0.095, p=0.84	r=0.476, p=0.243	r=-0.762, p=0.037 *	r=0.595, p=0.132	r=0.595, p=0.132	r=0.179, p=0.713	r=0.024, p=0.977	r=0.25, p=0.595	r=0.476, p=0.243
**Zn**	r=0.405, p=0.327	r=0.095, p=0.84	r=-0.19, p=0.665	r=0.857, p=0.011 *	---	r=0.095, p=0.84	r=0.714, p=0.058	r=-0.81, p=0.022 *	r=0.619, p=0.115	r=0.833, p=0.015 *	r=0.25, p=0.595	r=-0.286, p=0.501	r=0.286, p=0.556	r=0.405, p=0.327
**Fe**	r=0.119, p=0.793	r=-0.738, p=0.046 *	r=-0.595, p=0.132	r=-0.095, p=0.84	r=0.095, p=0.84	---	r=0.238, p=0.582	r=0.429, p=0.299	r=-0.476, p=0.243	r=0.238, p=0.582	r=0.357, p=0.444	r=-0.262, p=0.536	r=0.357, p=0.444	r=0.286, p=0.501
**Cu**	r=0.5, p=0.216	r=-0.048, p=0.935	r=-0.524, p=0.197	r=0.476, p=0.243	r=0.714, p=0.058	r=0.238, p=0.582	---	r=-0.333, p=0.428	r=0.19, p=0.665	r=0.786, p=0.028 *	r=0.679, p=0.11	r=-0.667, p=0.083	r=-0.357, p=0.444	r=0.357, p=0.389
**As**	r=-0.214, p=0.619	r=-0.429, p=0.299	r=-0.143, p=0.752	r=-0.762, p=0.037 *	r=-0.81, p=0.022 *	r=0.429, p=0.299	r=-0.333, p=0.428	---	r=-0.857, p=0.011 *	r=-0.5, p=0.216	r=0.071, p=0.906	r=0, p=1	r=-0.143, p=0.783	r=-0.071, p=0.882
**Se**	r=-0.238, p=0.582	r=0.69, p=0.069	r=0.405, p=0.327	r=0.595, p=0.132	r=0.619, p=0.115	r=-0.476, p=0.243	r=0.19, p=0.665	r=-0.857, p=0.011 *	---	r=0.286, p=0.501	r=-0.357, p=0.444	r=0, p=1	r=0.036, p=0.963	r=0.143, p=0.752
**Rb**	r=0.333, p=0.428	r=-0.19, p=0.665	r=-0.31, p=0.462	r=0.595, p=0.132	r=0.833, p=0.015 *	r=0.238, p=0.582	r=0.786, p=0.028 *	r=-0.5, p=0.216	r=0.286, p=0.501	---	r=0.679, p=0.11	r=-0.524, p=0.197	r=0.214, p=0.662	r=0.476, p=0.243
**Sr**	r=0.429, p=0.354	r=-0.214, p=0.662	r=-0.464, p=0.302	r=0.179, p=0.713	r=0.25, p=0.595	r=0.357, p=0.444	r=0.679, p=0.11	r=0.071, p=0.906	r=-0.357, p=0.444	r=0.679, p=0.11	---	r=-0.821, p=0.034 *	r=0.071, p=0.906	r=-0.143, p=0.783
**Mo**	r=-0.048, p=0.935	r=0.095, p=0.84	r=0.5, p=0.216	r=0.024, p=0.977	r=-0.286, p=0.501	r=-0.262, p=0.536	r=-0.667, p=0.083	r=0, p=1	r=0, p=1	r=-0.524, p=0.197	r=-0.821, p=0.034 *	---	r=0.143, p=0.783	r=0.167, p=0.703
**B**	r=-0.036, p=0.963	r=-0.321, p=0.498	r=0.071, p=0.906	r=0.25, p=0.595	r=0.286, p=0.556	r=0.357, p=0.444	r=-0.357, p=0.444	r=-0.143, p=0.783	r=0.036, p=0.963	r=0.214, p=0.662	r=0.071, p=0.906	r=0.143, p=0.783	---	r=-0.036, p=0.963

	Ca	Mg	Na	K	Zn	Fe	Cu	As	Se	Rb	Sr	Mo	B	Mn
**Mn**	r=0.024, p=0.977	r=0, p=1	r=0.214, p=0.619	r=0.476, p=0.243	r=0.405, p=0.327	r=0.286, p=0.501	r=0.357, p=0.389	r=-0.071, p=0.882	r=0.143, p=0.752	r=0.476, p=0.243	r=-0.143, p=0.783	r=0.167, p=0.703	r=-0.036, p=0.963	---

r - Spearman's correlation coefficient.

Table S3. Comparison of the correlation of 14 elements in the kidney of rats receiving the macrogenistein diet.

	Ca	Mg	Na	K	Zn	Fe	Cu	As	Se	Rb	Sr	Mo	B	Mn
**Ca**	---	r=-0.367, p=0.336	r=-0.77, p=0.015 *	r=0.283, p=0.463	r=0.55, p=0.133	r=-0.017, p=0.982	r=0.367, p=0.336	r=0.95, p<0.001 *	r=0.717, p=0.037 *	r=0.15, p=0.708	r=0.6, p=0.097	r=0.933, p=0.001 *	r=-0.017, p=0.982	r=0.483, p=0.194
**Mg**	r=-0.367, p=0.336	---	r=0.31, p=0.417	r=-0.65, p=0.067	r=0.533, p=0.148	r=-0.717, p=0.037 *	r=-0.967, p<0.001 *	r=-0.417, p=0.27	r=-0.017, p=0.982	r=-0.617, p=0.086	r=-0.767, p=0.021 *	r=-0.433, p=0.25	r=0.483, p=0.194	r=0.467, p=0.213
**Na**	r=-0.77, p=0.015 *	r=0.31, p=0.417	---	r=-0.184, p=0.635	r=-0.368, p=0.33	r=-0.126, p=0.748	r=-0.301, p=0.431	r=-0.653, p=0.057	r=-0.343, p=0.366	r=-0.067, p=0.864	r=-0.318, p=0.404	r=-0.569, p=0.11	r=-0.151, p=0.699	r=-0.167, p=0.667
**K**	r=0.283, p=0.463	r=-0.65, p=0.067	r=-0.184, p=0.635	---	r=-0.467, p=0.213	r=0.467, p=0.213	r=0.633, p=0.076	r=0.233, p=0.552	r=-0.183, p=0.644	r=0.817, p=0.011 *	r=0.733, p=0.031 *	r=0.217, p=0.581	r=-0.117, p=0.776	r=-0.533, p=0.148
**Zn**	r=0.55, p=0.133	r=0.533, p=0.148	r=-0.368, p=0.33	r=-0.467, p=0.213	---	r=-0.717, p=0.037 *	r=-0.533, p=0.148	r=0.5, p=0.178	r=0.667, p=0.059	r=-0.55, p=0.133	r=-0.2, p=0.613	r=0.483, p=0.194	r=0.233, p=0.552	r=0.933, p=0.001 *
**Fe**	r=-0.017, p=0.982	r=-0.717, p=0.037 *	r=-0.126, p=0.748	r=0.467, p=0.213	r=-0.717, p=0.037 *	---	r=0.783, p=0.017 *	r=-0.017, p=0.982	r=-0.233, p=0.552	r=0.667, p=0.059	r=0.533, p=0.148	r=0.017, p=0.982	r=-0.033, p=0.948	r=-0.683, p=0.05
**Cu**	r=0.367, p=0.336	r=-0.967, p<0.001 *	r=-0.301, p=0.431	r=0.633, p=0.076	r=-0.533, p=0.148	r=0.783, p=0.017 *	---	r=0.367, p=0.336	r=-0.017, p=0.982	r=0.583, p=0.108	r=0.717, p=0.037 *	r=0.4, p=0.291	r=-0.45, p=0.23	r=-0.5, p=0.178
**As**	r=0.95, p<0.001 *	r=-0.417, p=0.27	r=-0.653, p=0.057	r=0.233, p=0.552	r=0.5, p=0.178	r=-0.017, p=0.982	r=0.367, p=0.336	---	r=0.833, p=0.008 *	r=0.2, p=0.613	r=0.683, p=0.05	r=0.983, p<0.001 *	r=-0.033, p=0.948	r=0.533, p=0.148
**Se**	r=0.717, p=0.037 *	r=-0.017, p=0.982	r=-0.343, p=0.366	r=-0.183, p=0.644	r=0.667, p=0.059	r=-0.233, p=0.552	r=-0.017, p=0.982	r=0.833, p=0.008 *	---	r=0, p=1	r=0.417, p=0.27	r=0.85, p=0.006 *	r=0.233, p=0.552	r=0.8, p=0.014 *
**Rb**	r=0.15, p=0.708	r=-0.617, p=0.086	r=-0.067, p=0.864	r=0.817, p=0.011 *	r=-0.55, p=0.133	r=0.667, p=0.059	r=0.583, p=0.108	r=0.2, p=0.613	r=0, p=1	---	r=0.817, p=0.011 *	r=0.217, p=0.581	r=0.2, p=0.613	r=-0.483, p=0.194
**Sr**	r=0.6, p=0.097	r=-0.767, p=0.021 *	r=-0.318, p=0.404	r=0.733, p=0.031 *	r=-0.2, p=0.613	r=0.533, p=0.148	r=0.717, p=0.037 *	r=0.683, p=0.05	r=0.417, p=0.27	r=0.817, p=0.011 *	---	r=0.7, p=0.043 *	r=-0.05, p=0.912	r=-0.1, p=0.81
**Mo**	r=0.933, p=0.001 *	r=-0.433, p=0.25	r=-0.569, p=0.11	r=0.217, p=0.581	r=0.483, p=0.194	r=0.017, p=0.982	r=0.4, p=0.291	r=0.983, p<0.001 *	r=0.85, p=0.006 *	r=0.217, p=0.581	r=0.7, p=0.043 *	---	r=-0.083, p=0.843	r=0.55, p=0.133
**B**	r=-0.017, p=0.982	r=0.483, p=0.194	r=-0.151, p=0.699	r=-0.117, p=0.776	r=0.233, p=0.552	r=-0.033, p=0.948	r=-0.45, p=0.23	r=-0.033, p=0.948	r=0.233, p=0.552	r=0.2, p=0.613	r=-0.05, p=0.912	r=-0.083, p=0.843	---	r=0.167, p=0.678

	Ca	Mg	Na	K	Zn	Fe	Cu	As	Se	Rb	Sr	Mo	B	Mn
**Mn**	r=0.483, p=0.194	r=0.467, p=0.213	r=-0.167, p=0.667	r=-0.533, p=0.148	r=0.933, p=0.001 *	r=-0.683, p=0.05	r=-0.5, p=0.178	r=0.533, p=0.148	r=0.8, p=0.014 *	r=-0.483, p=0.194	r=-0.1, p=0.81	r=0.55, p=0.133	r=0.167, p=0.678	---

r - Spearman's correlation coefficient.

Table S4. Comparison of the correlation of 14 elements in the kidney of rats receiving the microgenistein diet.

	Ca	Mg	Na	K	Zn	Fe	Cu	As	Se	Rb	Sr	Mo	B	Mn
**Ca**	---	r=0.417, p=0.27	r=0.2, p=0.613	r=0.583, p=0.108	r=0.717, p=0.037 *	r=-0.3, p=0.437	r=-0.75, p=0.025 *	r=0.483, p=0.194	r=-0.517, p=0.162	r=0.7, p=0.043 *	r=0.9, p=0.002 *	r=-0.133, p=0.744	r=0.45, p=0.23	r=0.533, p=0.148
**Mg**	r=0.417, p=0.27	---	r=-0.633, p=0.076	r=-0.167, p=0.678	r=0.783, p=0.017 *	r=-0.833, p=0.008 *	r=-0.133, p=0.744	r=0.85, p=0.006 *	r=0.35, p=0.359	r=0.617, p=0.086	r=0.417, p=0.27	r=0.667, p=0.059	r=0.017, p=0.982	r=0.633, p=0.076
**Na**	r=0.2, p=0.613	r=-0.633, p=0.076	---	r=0.6, p=0.097	r=-0.183, p=0.644	r=0.467, p=0.213	r=-0.467, p=0.213	r=-0.617, p=0.086	r=-0.85, p=0.006 *	r=-0.15, p=0.708	r=0.1, p=0.81	r=-0.583, p=0.108	r=0.233, p=0.552	r=-0.283, p=0.463
**K**	r=0.583, p=0.108	r=-0.167, p=0.678	r=0.6, p=0.097	---	r=0.3, p=0.437	r=0.033, p=0.948	r=-0.9, p=0.002 *	r=-0.133, p=0.744	r=-0.6, p=0.097	r=0.217, p=0.581	r=0.6, p=0.097	r=-0.383, p=0.312	r=0.633, p=0.076	r=0.367, p=0.336
**Zn**	r=0.717, p=0.037 *	r=0.783, p=0.017 *	r=-0.183, p=0.644	r=0.3, p=0.437	---	r=-0.683, p=0.05	r=-0.5, p=0.178	r=0.7, p=0.043 *	r=0, p=1	r=0.767, p=0.021 *	r=0.617, p=0.086	r=0.55, p=0.133	r=0.45, p=0.23	r=0.833, p=0.008 *
**Fe**	r=-0.3, p=0.437	r=-0.833, p=0.008 *	r=0.467, p=0.213	r=0.033, p=0.948	r=-0.683, p=0.05	---	r=0.133, p=0.744	r=-0.817, p=0.011 *	r=-0.417, p=0.27	r=-0.367, p=0.336	r=-0.283, p=0.463	r=-0.75, p=0.025 *	r=-0.017, p=0.982	r=-0.567, p=0.121
**Cu**	r=-0.75, p=0.025 *	r=-0.133, p=0.744	r=-0.467, p=0.213	r=-0.9, p=0.002 *	r=-0.5, p=0.178	r=0.133, p=0.744	---	r=-0.1, p=0.81	r=0.617, p=0.086	r=-0.483, p=0.194	r=-0.767, p=0.021 *	r=0.317, p=0.41	r=-0.617, p=0.086	r=-0.533, p=0.148
**As**	r=0.483, p=0.194	r=0.85, p=0.006 *	r=-0.617, p=0.086	r=-0.133, p=0.744	r=0.7, p=0.043 *	r=-0.817, p=0.011 *	r=-0.1, p=0.81	---	r=0.433, p=0.25	r=0.683, p=0.05	r=0.533, p=0.148	r=0.583, p=0.108	r=-0.067, p=0.88	r=0.683, p=0.05
**Se**	r=-0.517, p=0.162	r=0.35, p=0.359	r=-0.85, p=0.006 *	r=-0.6, p=0.097	r=0, p=1	r=-0.417, p=0.27	r=0.617, p=0.086	r=0.433, p=0.25	---	r=-0.083, p=0.843	r=-0.4, p=0.291	r=0.7, p=0.043 *	r=-0.267, p=0.493	r=0.217, p=0.581
**Rb**	r=0.7, p=0.043 *	r=0.617, p=0.086	r=-0.15, p=0.708	r=0.217, p=0.581	r=0.767, p=0.021 *	r=-0.367, p=0.336	r=-0.483, p=0.194	r=0.683, p=0.05	r=-0.083, p=0.843	---	r=0.8, p=0.014 *	r=0.217, p=0.581	r=0.117, p=0.776	r=0.8, p=0.014 *
**Sr**	r=0.9, p=0.002 *	r=0.417, p=0.27	r=0.1, p=0.81	r=0.6, p=0.097	r=0.617, p=0.086	r=-0.283, p=0.463	r=-0.767, p=0.021 *	r=0.533, p=0.148	r=-0.4, p=0.291	r=0.8, p=0.014 *	---	r=-0.183, p=0.644	r=0.233, p=0.552	r=0.617, p=0.086
**Mo**	r=-0.133, p=0.744	r=0.667, p=0.059	r=-0.583, p=0.108	r=-0.383, p=0.312	r=0.55, p=0.133	r=-0.75, p=0.025 *	r=0.317, p=0.41	r=0.583, p=0.108	r=0.7, p=0.043 *	r=0.217, p=0.581	r=-0.183, p=0.644	---	r=-0.05, p=0.912	r=0.467, p=0.213
**B**	r=0.45, p=0.23	r=0.017, p=0.982	r=0.233, p=0.552	r=0.633, p=0.076	r=0.45, p=0.23	r=-0.017, p=0.982	r=-0.617, p=0.086	r=-0.067, p=0.88	r=-0.267, p=0.493	r=0.117, p=0.776	r=0.233, p=0.552	r=-0.05, p=0.912	---	r=0.45, p=0.23

	Ca	Mg	Na	K	Zn	Fe	Cu	As	Se	Rb	Sr	Mo	B	Mn
**Mn**	r=0.533, p=0.148	r=0.633, p=0.076	r=-0.283, p=0.463	r=0.367, p=0.336	r=0.833, p=0.008 *	r=-0.567, p=0.121	r=-0.533, p=0.148	r=0.683, p=0.05	r=0.217, p=0.581	r=0.8, p=0.014 *	r=0.617, p=0.086	r=0.467, p=0.213	r=0.45, p=0.23	---

r - Spearman's correlation coefficient. * statistically significant ($p < 0.05$).

Table S5. Comparison of the correlation of 14 elements in the kidney of rats receiving the nanogenistein diet.

	Ca	Mg	Na	K	Zn	Fe	Cu	As	Se	Rb	Sr	Mo	B	Mn
**Ca**	---	r=-0.412, p=0.271	r=0.904, p=0.001 *	r=0.946, p<0.001 *	r=-0.367, p=0.336	r=0.483, p=0.194	r=0.483, p=0.194	r=-0.467, p=0.213	r=-0.533, p=0.148	r=-0.483, p=0.194	r=0.317, p=0.41	r=0.8, p=0.014 *	r=-0.45, p=0.23	r=0.583, p=0.108
**Mg**	r=-0.412, p=0.271	---	r=-0.384, p=0.308	r=-0.401, p=0.285	r=-0.412, p=0.271	r=-0.891, p=0.001 *	r=-0.941, p<0.001 *	r=-0.513, p=0.158	r=0.748, p=0.02 *	r=0.916, p=0.001 *	r=-0.924, p<0.001 *	r=-0.597, p=0.09	r=0.866, p=0.003 *	r=-0.723, p=0.028 *
**Na**	r=0.904, p=0.001 *	r=-0.384, p=0.308	---	r=0.95, p<0.001 *	r=-0.427, p=0.252	r=0.477, p=0.194	r=0.41, p=0.273	r=-0.502, p=0.168	r=-0.644, p=0.061	r=-0.427, p=0.252	r=0.293, p=0.444	r=0.686, p=0.041 *	r=-0.527, p=0.145	r=0.611, p=0.081
**K**	r=0.946, p<0.001 *	r=-0.401, p=0.285	r=0.95, p<0.001 *	---	r=-0.427, p=0.252	r=0.502, p=0.168	r=0.444, p=0.232	r=-0.527, p=0.145	r=-0.644, p=0.061	r=-0.502, p=0.168	r=0.243, p=0.529	r=0.695, p=0.038 *	r=-0.494, p=0.177	r=0.577, p=0.104
**Zn**	r=-0.367, p=0.336	r=-0.412, p=0.271	r=-0.427, p=0.252	r=-0.427, p=0.252	---	r=0.5, p=0.178	r=0.467, p=0.213	r=0.9, p=0.002 *	r=-0.267, p=0.493	r=-0.45, p=0.23	r=0.5, p=0.178	r=0.033, p=0.948	r=-0.483, p=0.194	r=0.3, p=0.437
**Fe**	r=0.483, p=0.194	r=-0.891, p=0.001 *	r=0.477, p=0.194	r=0.502, p=0.168	r=0.5, p=0.178	---	r=0.917, p=0.001 *	r=0.4, p=0.291	r=-0.85, p=0.006 *	r=-0.95, p<0.001 *	r=0.833, p=0.008 *	r=0.617, p=0.086	r=-0.933, p=0.001 *	r=0.783, p=0.017 *
**Cu**	r=0.483, p=0.194	r=-0.941, p<0.001 *	r=0.41, p=0.273	r=0.444, p=0.232	r=0.467, p=0.213	r=0.917, p=0.001 *	---	r=0.467, p=0.213	r=-0.7, p=0.043 *	r=-0.967, p<0.001 *	r=0.833, p=0.008 *	r=0.583, p=0.108	r=-0.883, p=0.003 *	r=0.667, p=0.059
**As**	r=-0.467, p=0.213	r=-0.513, p=0.158	r=-0.502, p=0.168	r=-0.527, p=0.145	r=0.9, p=0.002 *	r=0.4, p=0.291	r=0.467, p=0.213	---	r=-0.2, p=0.613	r=-0.4, p=0.291	r=0.583, p=0.108	r=-0.033, p=0.948	r=-0.433, p=0.25	r=0.233, p=0.552
**Se**	r=-0.533, p=0.148	r=0.748, p=0.02 *	r=-0.644, p=0.061	r=-0.644, p=0.061	r=-0.267, p=0.493	r=-0.85, p=0.006 *	r=-0.7, p=0.043 *	r=-0.2, p=0.613	---	r=0.767, p=0.021 *	r=-0.65, p=0.067	r=-0.633, p=0.076	r=0.917, p=0.001 *	r=-0.867, p=0.005 *
**Rb**	r=-0.483, p=0.194	r=0.916, p=0.001 *	r=-0.427, p=0.252	r=-0.502, p=0.168	r=-0.45, p=0.23	r=-0.95, p<0.001 *	r=-0.967, p<0.001 *	r=-0.4, p=0.291	r=0.767, p=0.021 *	---	r=-0.783, p=0.017 *	r=-0.583, p=0.108	r=0.883, p=0.003 *	r=-0.7, p=0.043 *
**Sr**	r=0.317, p=0.41	r=-0.924, p<0.001 *	r=0.293, p=0.444	r=0.243, p=0.529	r=0.5, p=0.178	r=0.833, p=0.008 *	r=0.833, p=0.008 *	r=0.583, p=0.108	r=-0.65, p=0.067	r=-0.783, p=0.017 *	---	r=0.65, p=0.067	r=-0.783, p=0.017 *	r=0.767, p=0.021 *
**Mo**	r=0.8, p=0.014 *	r=-0.597, p=0.09	r=0.686, p=0.041 *	r=0.695, p=0.038 *	r=0.033, p=0.948	r=0.617, p=0.086	r=0.583, p=0.108	r=-0.033, p=0.948	r=-0.633, p=0.076	r=-0.583, p=0.108	r=0.65, p=0.067	---	r=-0.583, p=0.108	r=0.883, p=0.003 *
**B**	r=-0.45, p=0.23	r=0.866, p=0.003 *	r=-0.527, p=0.145	r=-0.494, p=0.177	r=-0.483, p=0.194	r=-0.933,	r=-0.883,	r=-0.433, p=0.25	r=0.917, p=0.001 *	r=0.883, p=0.003 *	r=-0.783,	r=-0.583, p=0.108	---	r=-0.817,

	Ca	Mg	Na	K	Zn	Fe	Cu	As	Se	Rb	Sr	Mo	B	Mn
						p=0.001 *	p=0.003 *				p=0.017 *			p=0.011 *
**Mn**	r=0.583, p=0.108	r=-0.723, p=0.028 *	r=0.611, p=0.081	r=0.577, p=0.104	r=0.3, p=0.437	r=0.783, p=0.017 *	r=0.667, p=0.059	r=0.233, p=0.552	r=-0.867, p=0.005 *	r=-0.7, p=0.043 *	r=0.767, p=0.021 *	r=0.883, p=0.003 *	r=-0.817, p=0.011 *	---

r - Spearman's correlation coefficient. * statistically significant ($p < 0.05$).
